# Comparative compositional analysis of commercially produced mule duck and Landes goose foie gras

**DOI:** 10.1016/j.psj.2026.107681

**Published:** 2026-08-23

**Authors:** Yutong Xiao, Qiming Mu, Dapeng Liu, Hehe Tang, Hongfei Liu, He Zhang, Sirui Liu, Sidi Zhang, Jiaxin Zhang, Yongfu Zhang, Lipeng Yuan, Zhichao Hu, Zhanbao Guo, Yi Tang, Shuisheng Hou, Jun Zhang, Wei Min, Zhengkui Zhou

**Affiliations:** a State Key Laboratory of Animal Biotech Breeding, Institute of Animal Science, Chinese Academy of Agricultural Sciences, Beijing 100193, China; b Chinese Meat Duck Germplasm Innovation Center (Xian County), Xian County, Hebei Province, 062250, China; c Jilin Zhengfang Animal Husbandry Co., Ltd., Meihekou City, China

**Keywords:** Foie gras, Mule duck, Landes goose, SPME-GC-MS, Metabolomics

## Abstract

This study compared the compositional characteristics of commercially produced foie gras from mule ducks and Landes geese by integrating solid-phase microextraction gas chromatography-mass spectrometry, untargeted metabolomics, and untargeted lipidomics. Landes goose foie gras showed a significantly greater liver weight and higher moisture content than mule duck foie gras, indicating clear product-level differences in gross traits. Volatile profiling identified 153 compounds, of which 48 differed significantly between the two groups. Mule duck foie gras displayed a volatile profile enriched in floral-, fruity-, and spice-associated compounds, whereas Landes goose foie gras was characterized by a higher abundance of compounds associated with grassy, earthy, and lipid oxidation-related notes. Untargeted metabolomics identified 834 polar metabolites, including 132 differential metabolites, suggesting additional but relatively moderate compositional divergence at the small-molecule level. Untargeted lipidomics detected 536 lipids, among which 85 differed significantly. Mule duck foie gras was characterized by a higher relative abundance of several unsaturated triglycerides and selected phospholipid species, whereas Landes goose foie gras showed enrichment of several long-chain saturated triglycerides and bile-related components. Collectively, these results demonstrate that mule duck and Landes goose foie gras can be clearly differentiated at the levels of product traits, volatile composition, polar metabolites, and lipid profiles. The observed differences are best interpreted as integrated compositional differences between two commercially produced foie gras types obtained under species-specific production regimens, and they provide a useful basis for quality evaluation, product discrimination, and further targeted studies of flavor and lipid deposition patterns.

## Introduction

Foie gras is a traditional high-value product obtained from waterfowl through controlled overfeeding, during which the liver undergoes marked steatosis and reaches a commercially desirable size and texture. In modern production systems, mule ducks have become the dominant raw material for foie gras manufacture, whereas goose foie gras, particularly that derived from Landes geese, remains an important premium product category (Liu et al., 2016). Despite their shared commercial identity as foie gras, these products are derived from different waterfowl types and are commonly produced under different overfeeding regimens, which may contribute to substantial differences in physicochemical properties and compositional characteristics.

Previous studies on foie gras have mainly focused on hepatic steatosis mechanisms, overfeeding kinetics, technological yield, and selected quality traits such as liver weight, dry matter, lipid deposition, and processing loss (Lo et al., 2022; Yu et al., 2024) . Existing work has shown that overfeeding duration and metabolic stage can strongly influence foie gras composition, indicating that foie gras should be considered a dynamic product rather than a static endpoint (Arroyo et al., 2013; Bonnefont et al., 2019; Tavernier et al., 2020). At the same time, the chemical basis underlying product-to-product differences between commercially relevant foie gras types has not been fully characterized, especially from an integrated flavor and lipid composition perspective.

Flavor is one of the most important quality dimensions of foie gras, and volatile compounds are central to aroma differentiation among products. In parallel, small-molecule metabolites and lipid species contribute to compositional identity and may reflect differences in lipid deposition pattern, metabolic status, and structural composition (Lu et al., 2021). Untargeted metabolomics and lipidomics have increasingly been used in food research to characterize subtle product differences beyond conventional proximate analysis. For foie gras, such approaches may provide a more systematic understanding of how different commercially produced products diverge at the levels of aroma-related compounds, polar metabolites, and lipid classes.

Mule duck foie gras and Landes goose foie gras differ not only in species background but also, in practice, in body size, overfeeding duration, and production rhythm. These factors may jointly shape the final product. Therefore, a compositional comparison between the two has value not only for quality evaluation, but also for product discrimination and the identification of candidate chemical markers associated with different foie gras types.

In the present study, we compared commercially produced mule duck foie gras and Landes goose foie gras using gross trait measurements, SPME-GC-MS, untargeted metabolomics, and untargeted lipidomics. The aim was to characterize differences in liver traits, volatile compounds, polar metabolites, and lipid composition, and to provide a chemical basis for evaluating and distinguishing these two foie gras types.

## Materials and methods

### Animal experiment

Commercial male mule ducks (n = 13) and male Landes geese (n = 13) were provided by Zhengfang Animal Husbandry Co., Ltd. The mule ducks were produced by artificial insemination of male Muscovy ducks and female Peking ducks and were derived from the same batch under consistent hatching conditions. The Landes geese were also obtained from the same batch with uniform hatching conditions. During the growing period before overfeeding, all birds were reared on wire-mesh floors with ad libitum access to feed and water.

Mule ducks were transferred to individual cages and subjected to overfeeding from 68 days of age for 18 days. Their average body weight before slaughter was 5.75 kg. Landes geese were subjected to overfeeding from 65 days of age for 29 days, and their average body weight before slaughter was 7.70 kg. Before overfeeding, all birds were fed a conventional diet. During the overfeeding period, mule ducks and Landes geese received corn-based diets formulated according to their respective commercial production protocols. The ingredient composition and calculated nutrient levels of the overfeeding diets are provided below on an as-fed basis (Table S1).

At the end of the overfeeding period, birds were slaughtered by exsanguination after suspension. The livers were immediately excised after evisceration. Approximately 100 g of tissue was collected from the middle portion of the large liver lobe, rapidly frozen on dry ice, transported under frozen conditions, and stored at −80°C until analysis. All analyses were performed at the Central Laboratory of the Institute of Animal Science and Veterinary Medicine, Chinese Academy of Agricultural Sciences.

### Freeze-drying treatment

For moisture determination and subsequent compositional comparison, liver samples were freeze-dried using a vacuum freeze dryer. The system was precooled for at least 2 h before use. Samples were pre-frozen prior to loading and wrapped with aluminum foil to minimize deformation during drying. The samples were lyophilized for 36 h and then further dried in a desiccator for an additional 36 h before weighing analysis.

### Volatile compound analysis by solid phase microextraction-gas chromatography-mass spectrometry (SPME-GC-MS)

Volatile compounds in heated liver samples were determined using SPME-GC-MS. Before extraction, frozen raw liver samples were ground into powder in liquid nitrogen. 3 g of each sample was placed in a 20 mL headspace vial, and 10 µL of 2-methyl-3-heptanone (0.1 µg/µL) was added as an internal standard. Then sealed the vials immediately and heated at 85°C for 15 min (standardized cooking treatment) and equilibrated at 55°C for 20 min. Headspace extraction was performed at 55°C for 40 min using a 50/30 µm DVB/CAR/PDMS fiber (Supelco, USA). After extraction, the fiber was desorbed in the GC inlet at 250°C for 3 min. Data analysis was conducted on a Q-Exactive Orbitrap mass spectrometer (Thermo Fisher Scientific, USA) equipped with a TriPlus RSH autosampler and a Trace 1310 GC.

Chromatographic separation was carried out on a VF-WAX ms capillary column (60 m × 0.25 mm i.d., film thickness 0.25 µm). The oven was maintained at 40°C for 2 min initially, then ramped up to 230°C at 4°C/min and kept at the final temperature for 5 min. High-purity helium served as the carrier gas with a constant flow rate of 1 mL/min. The mass spectrometer was operated under electron impact (EI) ionization at 70 eV. The transfer line temperature was set to 250°C, and the automatic gain control (AGC) was set to a target of 1 × 10⁶. Full-scan mode was applied over the m/z range of 30 to 400 at a resolution of 60,000.

Two blank tests and two fiber conditioning runs were conducted before sample injection to eliminate residual contaminants. A quality control (QC) sample was tested every 14 injections to evaluate instrument stability throughout the analysis. Compound identification was performed with strict criteria: HRF score > 95 and MS match factor > 750. The retention index (RI) deviation was limited within 20 for the self-established database and below 50 for the NIST database.

### Metabolite profiling by LC-MS-based untargeted metabolomics

Untargeted metabolomic profiling of metabolites was performed using an MTBE-based biphasic extraction procedure. Briefly, approximately 20 mg of frozen liver sample was weighed into a 2-mL Eppendorf tube after grinding under liquid nitrogen to minimize degradation. Then, 300 μL of methanol was added and the internal standards were prepared by dissolving the standards in methanol at the beginning of extraction. A 5-mm stainless steel bead was added, and the sample was homogenized using a high-throughput tissue homogenizer (Retsch GmbH, Haan, Germany) at 60 Hz and 10°C for 60 s.

After homogenization, the steel bead was removed magnetically. Subsequently, 1 mL of methyl tert‑butyl ether was added, followed by vortexing for 1 min using a vortex mixer. Then, 250 μL of deionized water was added to induce phase separation. The extraction mixture was subjected to three repeated cycles of vortexing for 1 min and standing for 2 min. After sufficient extraction, the samples were centrifuged at 8000 rpm for 15 min at 10°C using a high-speed refrigerated centrifuge to ensure complete separation of the upper and lower phases. Then, 250 μL of the lower phase was collected and dried to completeness under vacuum using a vacuum concentrator (Thermo Fisher Scientific, USA). The dried residue was then reconstituted and vortexed thoroughly. The resulting supernatant was transferred to a 2-mL LC/MS glass vial for ultra-high-pressure liquid chromatography (UHPLC)-MS analysis.QC samples were prepared by pooling equal aliquots from all metabolite extracts. The pooled QC sample was vortex-mixed thoroughly and aliquoted into four LC/MS vials before instrumental analysis.

LC-MS/MS analysis was performed on a Vanquish UHPLC system (Thermo Fisher Scientific, Waltham, MA, USA) coupled to a Q Exactive HF-X Hybrid Quadrupole-Orbitrap mass spectrometer (Thermo Fisher Scientific, Waltham, MA, USA). Chromatographic separation was achieved using a UPLC BEH Amide column (2.1 mm × 100 mm, 1.7 μm, Waters Corporation, Milford, MA, USA). The acquisition software continuously evaluated full-scan MS spectra and triggered MS/MS acquisition in an information-dependent acquisition mode under the control of Xcalibur software (version 4.4, Thermo Fisher Scientific, USA). Raw data from positive and negative ion modes were processed separately. Metabolite annotation was performed based on the qualitative results of the corresponding metabolites.

### Lipid profiling by LC-MS-based untargeted lipidomics

Untargeted lipidomic profiling was performed using the same MTBE-based biphasic extraction procedure. Briefly, approximately 20 mg of frozen liver sample was ground under liquid nitrogen and transferred to a 2-mL Eppendorf tube. A volume of 300 μL methanol, or methanol containing isotopically labeled internal standards, was added first. For internal standard preparation, phospholipid standards were prepared at 2 ppm, and glyceride/fatty acid standards were prepared at 1–2 ppm. The standards were first diluted to 100 ppm in dichloromethane:methanol (1:1, v/v) and then further diluted to 1 ppm with methanol before use. The sample was homogenized with a 5-mm steel bead at 60 Hz and 10°C for 60 s.

After removal of the steel bead, 1 mL MTBE was added, and the sample was vortexed for 1 min. Then, 250 μL deionized water was added for phase separation. The extraction mixture underwent three repeated cycles of vortexing for 1 min followed by standing for 2 min. Samples were then centrifuged at 8000 rpm for 15 min at 10°C. The upper organic phase (950 μL) was transferred to a new 1.5-mL tube.

The collected upper organic phase was dried under a gentle stream of nitrogen until a transparent thin film was formed. Heating was avoided during solvent evaporation. The dried lipid extract was then reconstituted in 300 μL dichloromethane:methanol (1:1, v/v). The reconstituted extract was frozen overnight, followed by centrifugation at 14,000 rpm for 20 min at 4°C, and the supernatant was retained for analysis. The final extract was transferred into a transparent glass autosampler vial fitted with a glass insert for LC-MS analysis. According to the original analytical sequence, samples were first analyzed in negative ion mode.

QC samples were prepared by pooling 10–20 μL aliquots from each reconstituted lipid extract, followed by vortex mixing. The pooled QC sample was divided into four or more vials prior to analysis to minimize solvent loss caused by repeated opening.

Lipidomic analysis was performed on a Vanquish UHPLC system coupled to a Q Exactive HF-X Hybrid Quadrupole-Orbitrap mass spectrometer (Thermo Fisher Scientific, USA). The mass spectrometer was operated under Xcalibur software (version 4.4, Thermo Fisher Scientific, USA) in information-dependent acquisition mode. Raw data from positive and negative ion modes were processed separately. Lipid annotation was performed based on Metlin, DICP, MsDial_Meta, MoNA, MsDial_Lipid, GNPS, and HMDB, using accurate mass, isotope pattern, MS1 accurate mass and MS2 fragmentation patterns.

### Multivariate statistical analysis by OPLS-DA

Peak area data for volatile compounds, metabolites, and lipids were normalized according to the corresponding data-processing workflow and were then log-transformed and scaled before multivariate analysis. Orthogonal partial least squares-discriminant analysis (OPLS-DA) was performed using R (version 4.3.1) to visualize the separation of the relative-abundance profiles between mule duck and Landes goose foie gras. The OPLS-DA results were used as an exploratory multivariate visualization of group separation rather than as the sole criterion for defining differential compounds.

### Univariate statistical analysis and differential screening

For product traits and individual compounds, data normality was assessed using the Shapiro-Wilk test, and homogeneity of variance was evaluated using Levene's test. Variables satisfying the assumptions of normality and equal variance were compared between mule duck and Landes goose foie gras using a two-tailed Student's t-test. When the normality assumption was not met, the Mann-Whitney U test was used. For high-dimensional volatile, metabolomic, and lipidomic datasets, P values from pairwise comparisons were corrected for multiple testing using the Benjamini-Hochberg false discovery rate method, and the adjusted P value was reported as the q value. Fold change (FC) was calculated as the ratio of the mean normalized abundance between the two foie gras types, and log2(FC) was used to indicate the magnitude and direction of differences in volcano plots.

### Metabolic pathway enrichment analysis

Differential volatile compounds, differential metabolites, and differential lipids were identified using combined statistical significance and effect-size thresholds. Unless otherwise stated, compounds with |log2(FC)| ≥ 1 and q < 0.05 were considered differentially abundant. For pathway enrichment analysis, a stricter threshold of |log2(FC)| ≥ 1.5 and q < 0.05 was applied to obtain a more robust list of differential metabolites and lipids for input into MetaboAnalyst 6.0. Enriched pathways were analyzed separately according to the relative-abundance direction of the differential molecules. The enrichment results were interpreted as annotation-based pathway representation rather than direct evidence of metabolic flux or pathway activation.

## Results

### Liver performances and basic composition of mule duck and Landes goose foie gras

In interpreting these product-level differences, the unequal overfeeding duration between the two production systems should be considered as an important confounding factor. In the present study, mule ducks were overfed for 18 d, whereas Landes geese were overfed for 29 d, resulting in an 11-d longer overfeeding period for geese. Because overfeeding-induced hepatic steatosis is a time-dependent process, a longer overfeeding period may increase cumulative feed intake, hepatic enlargement, tissue hydration, triglyceride accumulation, lipid remodeling, and bile-related metabolic signatures. Therefore, the greater liver weight and some compositional features observed in Landes goose foie gras cannot be attributed solely to species or genetic background. Instead, these differences should be interpreted as the combined outcome of genetic background, body size, species-specific overfeeding tolerance, production rhythm, and cumulative overfeeding duration.

**Fig. 1:**
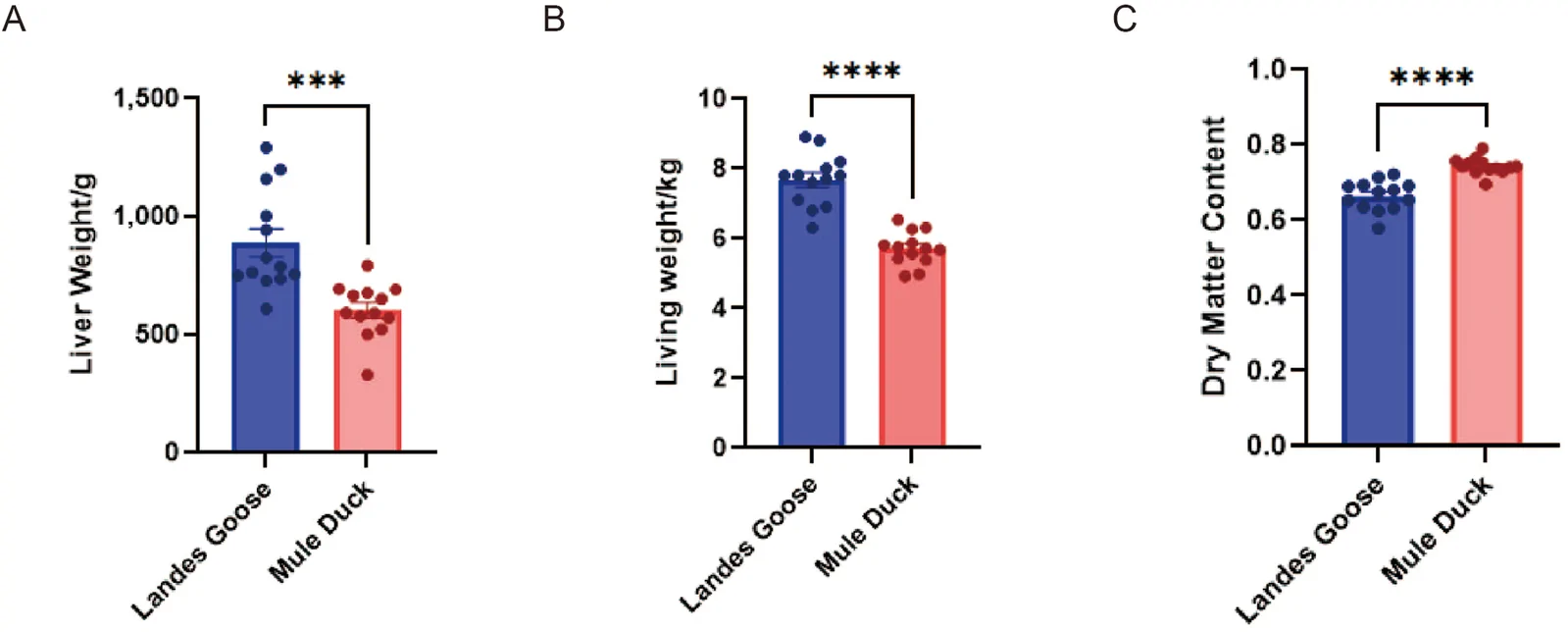
Production performance traits of foie gras from mule ducks and Landes geese.(A) Liver weight. (B) Body weight. (C) Liver-to-body weight ratio.

The foie gras produced by mule ducks and Landes geese had similar appearances, with smooth and soft textures, uniform pale yellow color both on the surface and interior, and inconspicuous blood vessels. However, clear differences were observed between mule duck foie gras and Landes goose foie gras at the quality trait level. The average weight of mule duck foie gras was 603 g, while that of Landes goose foie gras was 888 g, showing a statistically significant difference between the two groups. After vacuum freeze-drying, the moisture loss rates of the two foie gras samples also differed significantly. The average moisture content of mule duck foie gras was 66.47%, compared to 74.39% for Landes goose foie gras (Fig. 1).

These results indicate that the two foie gras products differed not only in liver size but also in compositional concentration. In practical terms, Landes goose foie gras appeared as a larger and more hydrated product, while mule duck foie gras showed a relatively more concentrated composition on a fresh-weight basis.

### Differential analysis of volatile flavor compounds and possible aromas

Orthogonal Partial Least Squares-Discriminant Analysis (**OPLS-DA**) showed significant and stable separation of volatile flavor compounds between mule duck foie gras and Landes goose foie gras. Obvious inter-group differences were observed, along with considerable intra-group variation among individual samples.

**Fig. 2:**
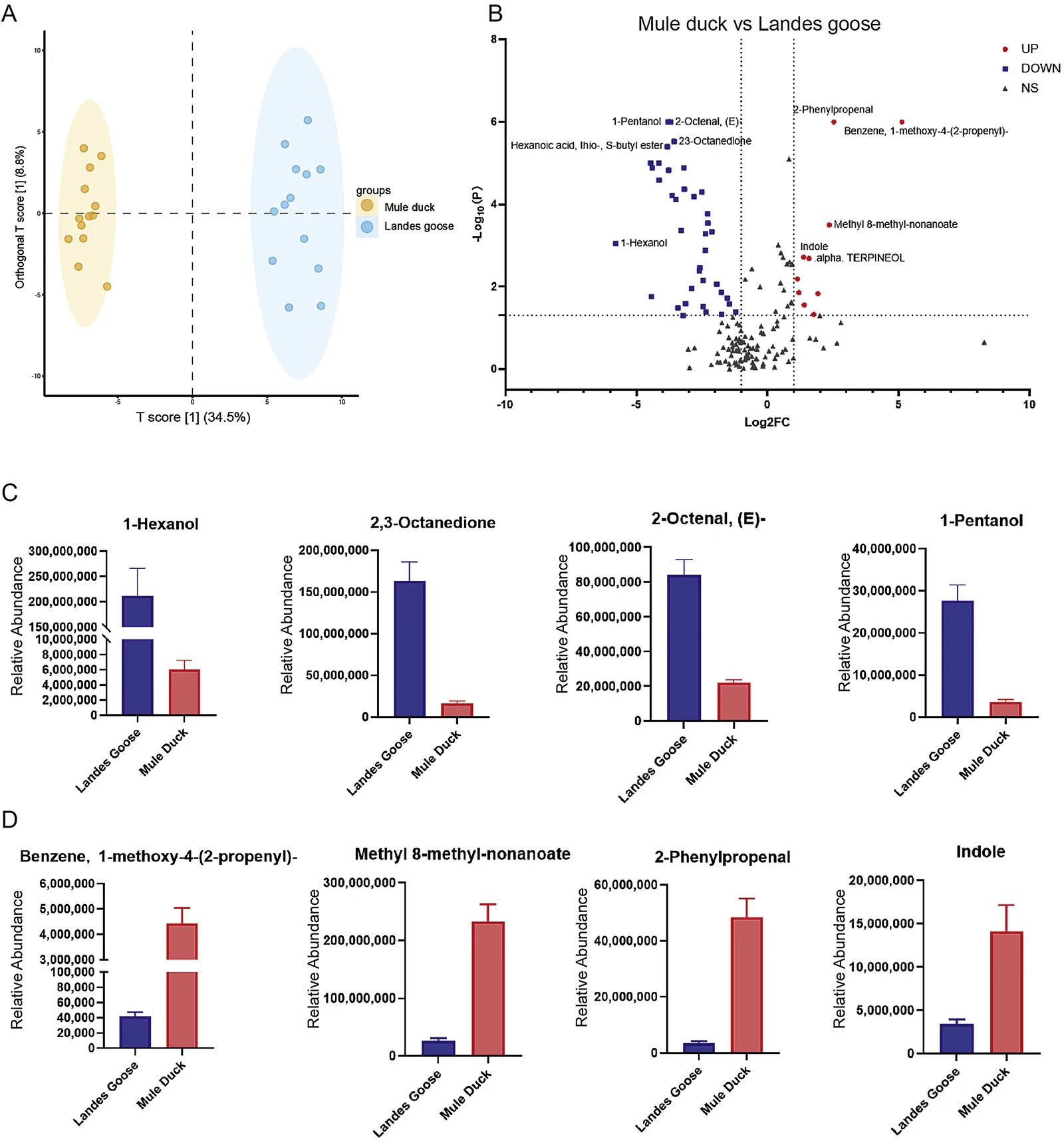
Differential analysis of volatile compounds in foie gras from mule ducks and Landes geese.(A) OPLS-DA score plot of differential volatile compounds between mule duck and Landes goose foie gras. (B) Volcano plot of differential volatile compounds between mule duck and Landes goose foie gras (|log2(FC)|≥1, q < 0.05). (C) The four most significantly enriched volatile compounds in Landes goose foie gras relative to mule duck foie gras. (D) The four most significantly enriched volatile compounds in mule duck foie gras relative to Landes goose foie gras.

A total of 153 volatile compounds were identified in this study, among which 48 showed significant differences in relative abundance between the two foie gras types (|log2(FC)| ≥ 1, q < 0.05) (Fig. S1). The relative-abundance profiles and literature-reported odor descriptors of these differential compounds are summarized (Fig. 2).

Among the volatile compounds with higher relative abundance in mule duck foie gras, representative compounds included benzene, 1‑methoxy-4-(2-propenyl)-(anethole), 1-(2-methoxypropoxy)-2-propanol, 2-phenylpropenal, methyl octanoate, and methyl 8-methylnonanoate (Alhusainy et al., 2012; Jashari et al., 2023; Starowicz and Granvogl, 2022). These compounds have been reported in the literature to be associated with floral, fruity, spice, and roasted odor descriptors. In Landes goose foie gras, compounds with higher relative abundance included pentanal, 2,3-octanedione, hexanal, 1-pentanol, and 1-hexanol (Lee and Lee, 2023; Li et al., 2022; Liu et al., 2022; Luo et al., 2022), which have been reported to be associated with green, grassy, mushroom-like, earthy, or lipid oxidation-related odor descriptors. Therefore, the volatile data suggest different odor-associated compositional profiles between the two foie gras products. However, because the present analysis measured relative volatile abundance rather than odor activity, these results cannot identify key aroma-active compounds or establish sensory intensity, sensory superiority, or consumer preference.

### Differential analysis of metabolites and potential functional properties

**Fig. 3:**
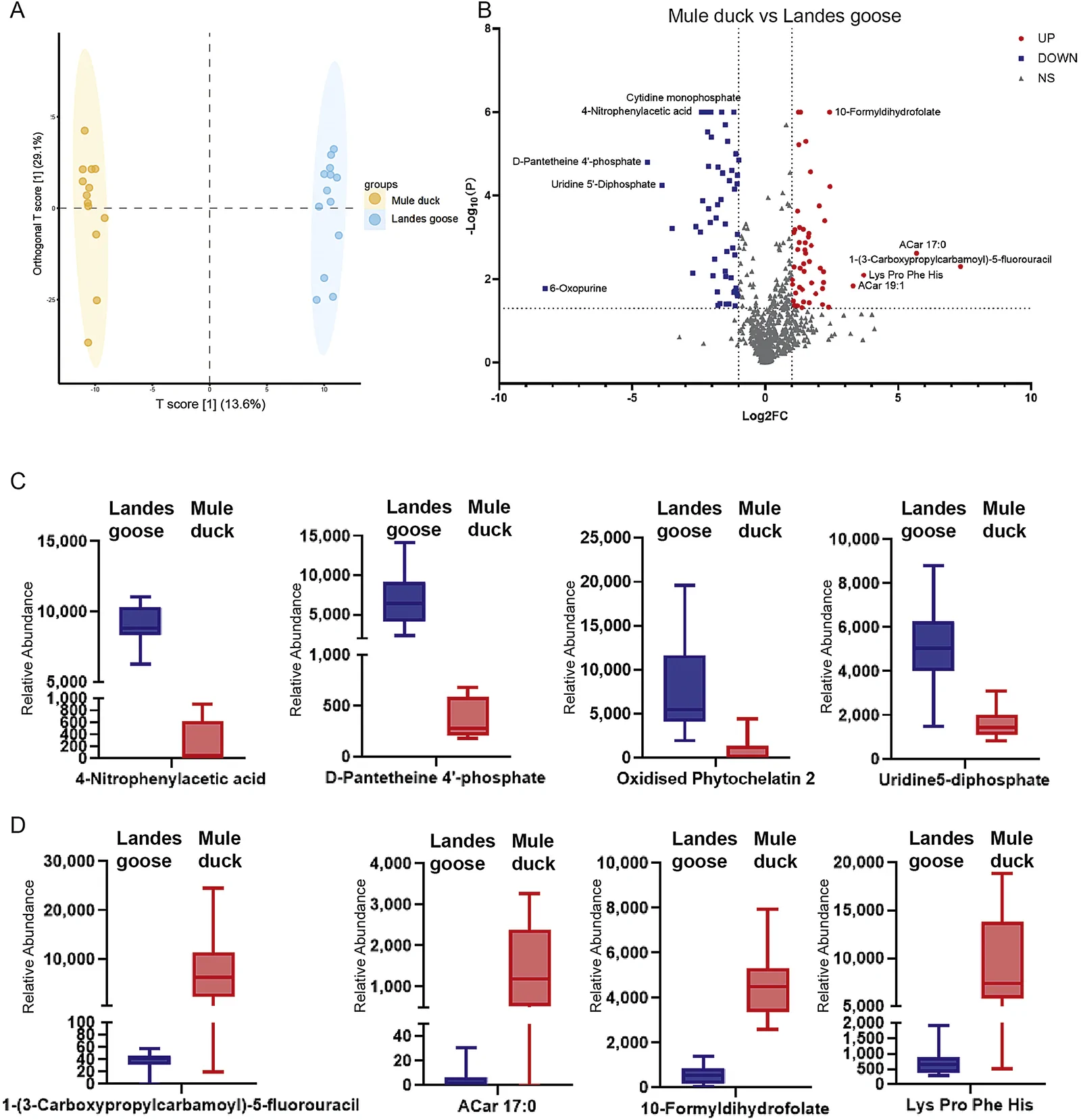
Differential metabolomic analysis of foie gras from mule ducks and Landes geese.(A) OPLS-DA score plot of differential metabolites between mule duck and Landes goose foie gras. (B) Volcano plot of differential metabolites between mule duck and Landes goose foie gras (|log2(FC)|≥1, q < 0.05). (C) The four most significantly enriched metabolites in Landes goose foie gras relative to mule duck foie gras. (D) The four most significantly enriched metabolites in mule duck foie gras relative to Landes goose foie gras.

Untargeted metabolomics identified 834 water-soluble metabolites, among which 132 showed significant differences in relative abundance between mule duck and Landes goose foie gras (|log2(FC)| ≥ 1, q < 0.05) (Fig. S2). These data demonstrate differences in relative metabolite composition between the two foie gras products; they do not by themselves establish taste intensity, physiological function, or nutritional benefit (Fig. 3).

Among the polar metabolites with higher relative abundance in mule duck foie gras, oligopeptides, stachyose/raffinose-family carbohydrates, cinnamic acid, and cinnamyl isovalerate were represented (Cinnamon, 2006; Ta et al., 2024). Several of these compounds have literature-reported taste descriptors; however, the present data do not establish sensory intensity. The mule duck-enriched profile also included NAD+/beta-NADH-related molecules, glutathione-related compounds, nucleotides, flavonoids, and polyphenols (De Boer and Boot, 1983; Lončar et al., 2020; Sanyal et al., 2023; Wang et al., 2022). These molecules have literature-reported roles in redox and intermediary metabolism (Pophaly et al., 2012; Wu et al., 2004), but their relative abundance in the samples does not demonstrate antioxidant activity, cell protection, anti-inflammatory effects, or other physiological benefits after consumption (Di Lorenzo et al., 2021). Landes goose foie gras showed higher relative abundance of tyrosine methyl ester, histidine methyl ester, coumarin, short-chain peptides, raffinose, inosine, nucleoside-related metabolites, phosphatidylethanolamine precursors, citicoline-related molecules, and proline betaine (Lloyd et al., 2011; Niemann et al., 2022). These findings indicate a distinct polar-metabolite composition rather than confirmed neural, metabolic, or sensory functions.

### Differential analysis of lipid composition, nutritional value, and potential functional properties

During the fattening process, the livers of waterfowl accumulate large amounts of fat. Therefore, lipids are the core components of foie gras, and their types and contents directly determine the nutritional characteristics, texture, and metabolic properties of foie gras, while participating in various physiological processes such as fat storage, cell membrane construction, and signal transduction. The main lipid classes detected in this study included: triglycerides (**TG**), diglycerides (**DG**), monogalactosyldiacylglycerols (**MGDG**), cholesterol esters (**CE**), phosphatidylcholines (**PC**), phosphatidylethanolamines (**PE**), sphingomyelins (**SM**), ceramides (**Cer**), hexosylceramides (**HexCer**), phosphatidylinositols (**PI)**, and phosphatidylglycerols (**PG**). 

**Fig. 4:**
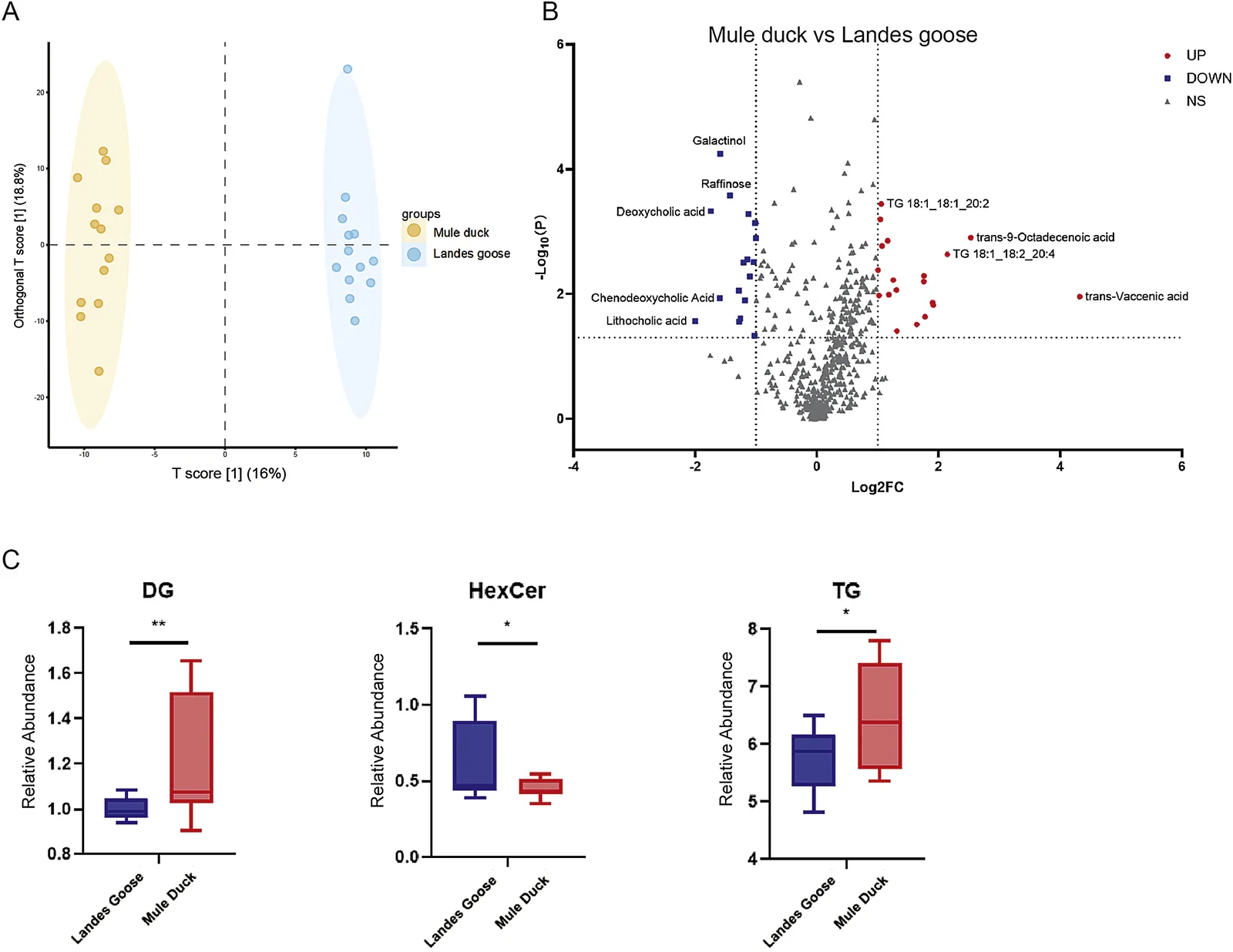
Differential lipidomic analysis of foie gras from mule ducks and Landes geese.(A) OPLS-DA score plot of differential lipids between mule duck and Landes goose foie gras. (B) Volcano plot of differential lipids between mule duck and Landes goose foie gras (|log2(FC)|≥1, q < 0.05). (C) Three lipid classes showing the most significant differences in abundance between mule duck and Landes goose foie gras.

Lipidomic differential analysis between mule duck and Landes goose foie gras showed significant differences in the relative abundance of diglycerides, hexosylceramides, and triglycerides among lipid classes, suggesting that these three lipid classes may serve as candidate compositional markers for distinguishing the two foie gras products. Untargeted lipidomics identified a total of 536 lipids, among which 85 showed significant differences in relative abundance (|log2(FC)| ≥ 1, q < 0.05) (Fig. S3). The differential lipids were concentrated in triglycerides, phospholipids, and sphingolipids (Fig. 4 and Table 1).

**Table 1:** Main differentially abundant lipids (|log2(FC)|≥3, q < 0.05).

Lipid	Average content in Landes goose	Average content in mule duck	log2(FC)
Alpha-Tocopherol	478.99	14553.37	4.93
Cer 18:1	389.68	6748.18	4.11
PC 24:0_ 20:3-OH(5)	293.51	4973.57	4.08
TG 15:0_18:4_20:5	458.18	6950.86	3.92
TG 18:1_18:1_20:3	152.84	2193.97	3.84
TG 18:1_18:2_20:4	73.17	921.35	3.65
TG 18:1_20:0_20:4	2022.60	21685.57	3.42
Cer 18:1	2643.61	330.28	−3.00
PE P-16:0_20:4	845.14	98.84	−3.10
PS(18:0_22:5)	2106.68	245.05	−3.10
PE 18:1_18:2	1286.57	130.40	−3.30
PC 20:4_22:5	15774.22	1557.85	−3.34
PG 18:0_18:2	823.19	75.35	−3.45
HexCer 22:1	3055.15	258.19	−3.56
Cer 18:1	1325.79	106.21	−3.64
MGDG O-13:1_18:1	7708.08	598.23	−3.69
PE 18:0_18:1	9399.45	677.97	−3.79
Cer(d18:1/24:1)	1858.17	111.41	−4.06
PI 18:0_18:1	7926.44	472.32	−4.07
PG 36:2	1626.28	96.58	−4.07
PE 16:0_18:0	1744.35	100.18	−4.12
PE O-20:4_16:1	1365.20	72.39	−4.24
PE 18:0_18:2	16335.38	732.10	−4.48
PC 16:0_18:1	92909.68	3408.57	−4.77
Lignoceric acid	3254.27	104.58	−4.96
Cer-NS d34:1	2912.10	78.41	−5.21
PE 16:0_18:1	15358.43	125.30	−6.94

TG is the main form of fat storage in animals, typically composed of 1 molecule of glycerol linked to 3 molecules of fatty acids via ester bonds, and is the core component of fat accumulation in foie gras (Jannasch et al., 2017). In lipidomic comparisons, differences in acyl-chain length, unsaturation degree, and double-bond composition can indicate differences in triglyceride structure and nutritional composition, but they cannot by themselves demonstrate health-promoting or pharmacological effects. The TG composition of mule duck foie gras showed a higher representation of medium-chain, unsaturated, and n-3 PUFA-related triglycerides, including TG 12:0_12:0_18:1, TG 15:0_18:4_20:5, and TG 22:6_18:1_18:1. These lipid species contain or imply fatty-acyl moieties such as stearidonic acid (SDA, 18:4n-3), eicosapentaenoic acid (EPA, 20:5n-3), docosahexaenoic acid (DHA, 22:6n-3), and odd-chain C15:0, among which EPA- and DHA-derived lipid mediators have been widely reported to participate in anti-inflammatory and pro-resolving pathways, DPA has been reported to downregulate pro-inflammatory factors in activated macrophages, and SDA can improve EPA status and EPA-derived oxylipin formation (Calder, 2015; Serhan et al., 2008; Seidel et al., 2024; Tian et al., 2017). In contrast, the TG profile of Landes goose foie gras was mainly represented by combinations of very-long-chain saturated fatty acids and monounsaturated fatty acids, such as TG 18:1_20:1_22:0 and TG 18:1_18:1_24:0, indicating a distinct long-chain and relatively more saturated TG structural pattern. This compositional pattern should not be interpreted as evidence of neural-tissue-related functionality or other direct physiological effects without targeted nutritional or biological validation .

In terms of phospholipid (PL) composition, the two foie gras types showed clear compositional differentiation. Mule duck foie gras was dominated by PE and phosphatidylglycerol (PG), with higher relative abundance of arachidonic acid-containing PE molecules and plasmalogen PE 20:4_P-16:0, accompanied by hydroxylated polyunsaturated fatty acid-modified PC 24:0_20:3-OH. These lipids may indicate greater representation of unsaturated phospholipid species and membrane lipid components with literature-reported antioxidant-associated characteristics, but they should not be interpreted as direct evidence of biological protection in consumers (Hansen and Artmann, 2008; Jacob et al., 2018; Luo et al., 2023). Landes goose foie gras possessed a broader phospholipid profile, with significant enrichment of PC, phosphatidylserine (PS), and PI. It was particularly rich in DHA-containing and hydroxylated DHA-type PC (PC 18:1_22:6 and PC 15:0_22:6-2OH) and highly unsaturated PS molecules. Because these lipid classes have been reported in relation to membrane structure and signal-related lipid biology, the Landes goose profile is described here as a structurally diverse phospholipid pattern rather than evidence of neural function or cognitive effects (Xiao and Chang, 2023; Zhou and Wei, 2023).

Differences in sphingolipids (SL) were concentrated in carbon-chain length and modification type. Mule duck foie gras showed higher relative abundance of very-long-chain ceramides, including Cer-NS d41:1 and Cer 18:1 (Cheng et al., 2021; Wang et al., 2021), whereas Landes goose foie gras was characterized by Cer-NS d34:1, Cer (d18:1/24:1), HexCer, and SM, with a higher representation of glycosphingolipids and saturated sphingomyelins (Cheng et al., 2021; Fan et al., 2024; Jiang et al., 2022). These findings indicate distinct sphingolipid compositions; they should not be interpreted as direct evidence of skin-barrier, cell-recognition, signal-regulation, metabolic-stress, or consumer health effects.

At the level of characteristic small molecules and fatty acids, mule duck foie gras was higher in alpha-tocopherol (vitamin E), natural trans vaccenic acid, and a small amount of elaidic acid, suggesting differences in antioxidant-related and fatty-acid compositional features. Landes goose foie gras was significantly enriched in very-long-chain saturated fatty acids (lignoceric acid, behenic acid, and arachidic acid), primary and secondary bile acids (deoxycholic acid, taurocholic acid, and chenodeoxycholic acid), functional amino acid-related molecules (taurine and l-norvaline), and various hydroxylated polyunsaturated phospholipids and ether phospholipids. These findings collectively indicate distinct lipid digestion-, membrane organization-, and small-molecule composition-related signatures between the two foie gras types. However, because this study only quantified molecular composition, these differences should be interpreted as compositional evidence with potential nutritional relevance rather than as proof of metabolic health benefits, neuroprotection, intestinal regulation, or pharmacological effects. (Table 2)

**Table 2:** Comparative biological and compositional features of mule duck and Landes goose foie gras.

Comparative dimension	Mule duck	Landes goose
Gross traits and product-level characteristics	Produces a smaller foie gras liver with a relatively more concentrated fresh-weight composition.	Produces a larger and more hydrated foie gras liver.
Triglyceride deposition pattern	Enriched in several unsaturated triglycerides, including TG 15:0_18:4_20:5, TG 18:1_18:1_20:3, TG 18:1_18:2_20:4, TG 18:1_20:0_20:4, and TG 22:6_18:1_18:1, indicating greater representation of polyunsaturated, odd-chain, and DHA/EPA-associated storage lipids. This profile is consistent with a more unsaturated neutral-lipid deposition pattern and suggests potential nutritional relevance.	More strongly represented by long-chain or very-long-chain saturated and monounsaturated triglyceride combinations, together with a broader bile-associated signature. Overall, the lipid pattern appears less concentrated on unsaturated TG enrichment and more oriented toward structural lipid diversity and high liver-mass deposition. However, no direct physiological function should be inferred from these compositional data alone.
Phospholipid and sphingolipid features	Characterized by higher abundance of selected phospholipid species such as PC 16:0_18:2, PE 18:1_20:4, PE 18:2_16:0, and hydroxylated PC 24:0_20:3-OH(5), together with ceramide-related species and high α-tocopherol. This pattern suggests stronger representation of unsaturated phospholipids and membrane lipid components with literature-reported antioxidant-associated characteristics.	Characterized by broader enrichment of structurally diverse membrane lipids, including PC 18:1_22:6, PC 15:0_22:6-2OH, PI 16:0_20:4, PE 18:0_22:6, PS species, SM, and HexCer. This profile indicates stronger representation of DHA-containing phospholipids, sphingolipid subclasses, and bile-associated lipid organization, but should not be interpreted as evidence of neural or cognitive effects.
Volatile and polar metabolite signatures	Volatile compounds with higher relative abundance included compounds with literature-reported floral-, fruity-, and spice-associated odor descriptors. These data indicate an odor-associated volatile compositional pattern, but not confirmed sensory intensity or aroma contribution. At the polar metabolite level, mule duck foie gras showed stronger representation of oligopeptides, stachyose/raffinose-family carbohydrates, oxidized glutathione, S-lactoylglutathione, NAD+, and acylcarnitine-related metabolites, suggesting stronger representation of redox buffering and intermediary metabolism.	Volatile profile enriched in aldehydes, alcohols, and ketones associated with grassy, earthy, mushroom-like, and lipid oxidation-related odor directions. At the polar metabolite level, Landes goose foie gras showed relatively greater representation of inosine, uridine-related metabolites, O-phosphorylethanolamine, and phospholipid precursor-associated compounds, suggesting broader nucleotide turnover and membrane precursor pools.
Pathway-level representation	Pathway enrichment of the mule duck-enriched differential set highlighted galactose metabolism, pyrimidine metabolism, phenylalanine metabolism, purine metabolism, glutathione metabolism, fatty acid degradation, taurine and hypotaurine metabolism, cysteine and methionine metabolism, and sphingolipid metabolism.	Pathway enrichment of the Landes goose-enriched differential set highlighted pantothenate and CoA biosynthesis, galactose metabolism, glycerophospholipid metabolism, and associated phosphatidylcholine/phosphatidylethanolamine-centered network organization.

This issue is particularly relevant to the interpretation of lipid accumulation patterns. Mule duck foie gras was obtained after a shorter 18-d overfeeding period and showed a relatively more concentrated fresh-weight composition with enrichment of several unsaturated triglycerides, whereas Landes goose foie gras was obtained after a longer 29-d overfeeding period and showed greater liver mass, higher moisture content, and enrichment of several long-chain saturated triglycerides and bile-related lipids. These differences may reflect distinct species-specific lipid deposition trajectories, but they may also reflect different stages of the overfeeding response. Without a time-matched design, a cumulative-feed-intake-matched design, or serial sampling at comparable steatosis stages, it is not possible to determine whether the observed differences in lipid accumulation are driven primarily by species genetic factors or by the 11-d difference in overfeeding duration.

In conclusion, although both mule duck foie gras and Landes goose foie gras are produced through overfeeding, clear product-level differences exist in their lipid deposition patterns and molecular composition. This study characterizes compositional differences between the two commercially produced foie gras types at the lipid molecular level, providing a scientific basis for quality evaluation, product discrimination, and future targeted assessment of their nutritional relevance.

### Pathway enrichment analysis of differential metabolites and lipids

**Fig. 5:**
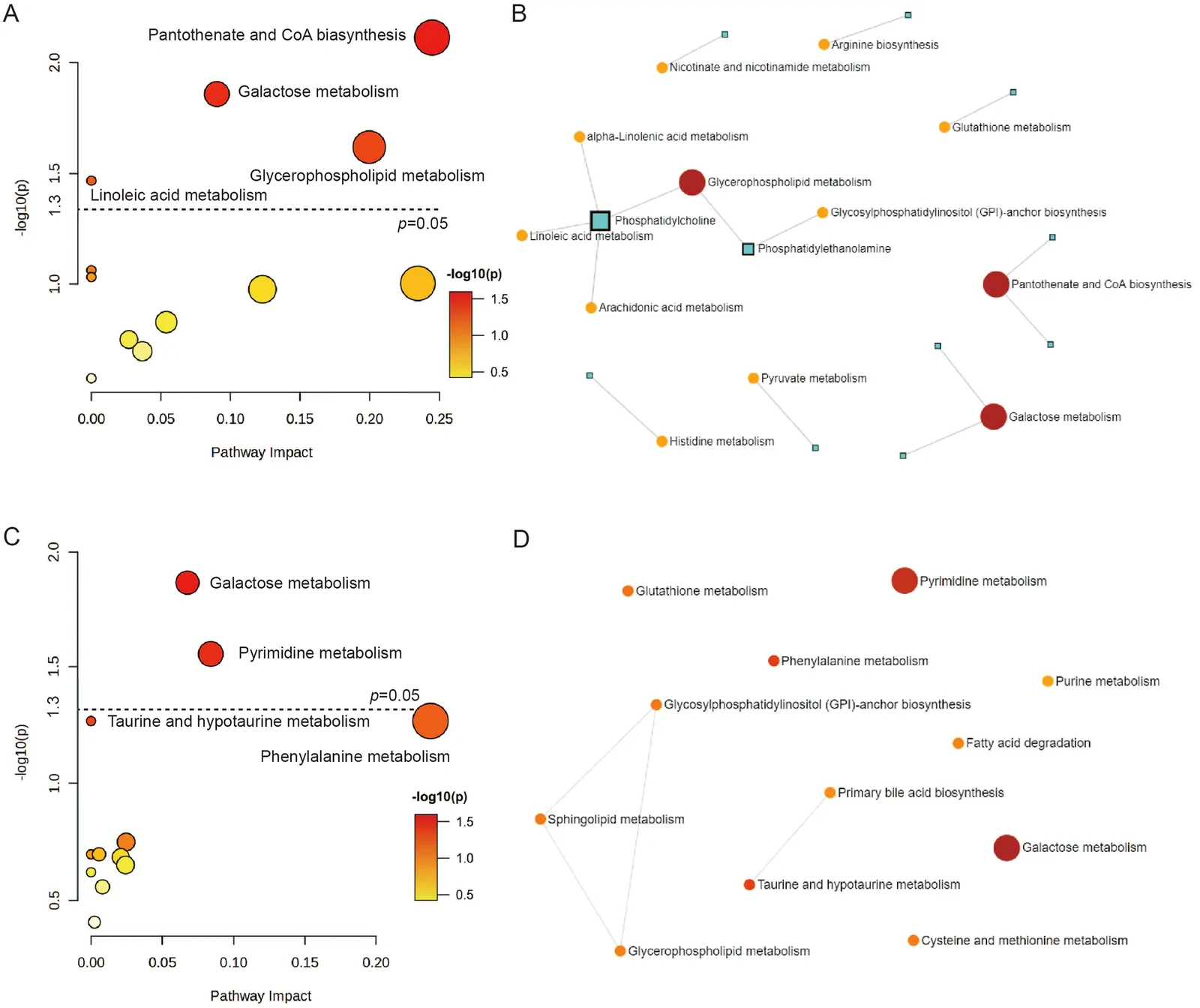
Pathway enrichment analysis of differentially abundant metabolites and lipids in foie gras from mule ducks and Landes geese. (A, B) Pathways enriched by metabolites and lipids with higher abundance in Landes goose foie gras (|log2(FC)|≥1.5, *p* < 0.05). (C, D) Pathways enriched by metabolites and lipids with higher abundance in mule duck foie gras (|log2(FC)|≥1.5, *p* < 0.05).

To further summarize the compositional divergence between mule duck and Landes goose foie gras at the pathway level, an integrated enrichment analysis was performed by jointly mapping the differentially abundant metabolites and lipids. For the compounds with higher abundance in Landes goose foie gras, the main enriched pathways included pantothenate and CoA biosynthesis, galactose metabolism, and glycerophospholipid metabolism. In the pathway interaction network, glycerophospholipid metabolism occupied a central position and was connected with phosphatidylcholine, phosphatidylethanolamine, alpha-linolenic acid metabolism, linoleic acid metabolism, and arachidonic acid metabolism. Additional associations were observed with nicotinate and nicotinamide metabolism, arginine biosynthesis, glutathione metabolism, glycosylphosphatidylinositol (**GPI**)-anchor biosynthesis, pyruvate metabolism, and histidine metabolism. Together, these results suggest that the Landes goose-enriched differential set was more strongly represented by phospholipid-related and cofactor-associated pathway annotations (Fig. 5).

For the compounds with higher abundance in mule duck foie gras, the major enriched pathways included galactose metabolism, pyrimidine metabolism, and phenylalanine metabolism. The corresponding pathway interaction network further involved purine metabolism, glutathione metabolism, fatty acid degradation, taurine and hypotaurine metabolism, cysteine and methionine metabolism, sphingolipid metabolism, glycerophospholipid metabolism, GPI-anchor biosynthesis, and primary bile acid biosynthesis. These findings indicate that the mule duck-enriched differential set showed broader representation in pathway annotations related to nucleotide metabolism, redox-associated metabolism, sulfur amino acid metabolism, and lipid-related metabolic processes.

## Discussion

This study provides an integrated comparison of commercially produced mule duck and Landes goose foie gras at the levels of gross traits, volatile compounds, polar metabolites, and lipids. Landes goose foie gras showed greater liver weight and higher moisture content than mule duck foie gras, whereas mule duck foie gras showed a relatively more concentrated compositional profile on a fresh-weight basis. This interpretation is consistent with previous studies showing that both breed and overfeeding procedure exert significant effects on key traits including liver weight and dry matter content during foie gras production (Bonnefont et al., 2019).

At the volatile level, mule duck and Landes goose foie gras were clearly separated. Mule duck foie gras was characterized by a volatile pattern enriched in floral-, fruity-, and spice-associated compounds, whereas Landes goose foie gras contained higher levels of compounds more commonly associated with grassy, earthy, mushroom-like, and lipid oxidation-related odor directions. These results indicate that the two foie gras types differ not only in the abundance of individual compounds, but also in the broader organization of their aroma-related chemical profiles. A cautious interpretation is that mule duck foie gras tends toward an ester- and aromatic compound-enriched pattern, whereas Landes goose foie gras tends toward an aldehyde-, alcohol-, and ketone-enriched pattern. Beyond their descriptive aroma roles, several of the differential volatile compounds may also be interpreted in relation to lipid oxidation chemistry. Aldehydes such as pentanal and hexanal, together with alcohols and ketones such as 1-pentanol, 1-hexanol, and 2,3-octanedione, have been widely reported as secondary products generated during the oxidation or degradation of unsaturated fatty acids in meat and other lipid-rich foods (Domínguez et al., 2019; Shahidi and Hossain, 2022). Thus, the higher abundance of these compounds in Landes goose foie gras may indicate a stronger lipid-oxidation-related volatile signature, whereas the ester- and aromatic-compound-enriched profile of mule duck foie gras may reflect a different balance between lipid-derived volatiles and non-lipid aroma contributors. However, because the present study relied on SPME-GC-MS without sensory validation, these findings should be regarded as compositional evidence for aroma differentiation rather than direct evidence of sensory superiority or consumer preference.

The metabolomic and lipidomic data further suggest that the two foie gras types differ in their underlying deposition patterns. Although the polar metabolite layer showed less decisive functional separation than the volatile and lipid layers, the presence of 132 differential metabolites still indicates measurable divergence in small-molecule composition. By contrast, lipidomics revealed a more structurally informative contrast. Mule duck foie gras showed a higher relative abundance of several unsaturated triglycerides and selected phospholipid species, while Landes goose foie gras was enriched in several long-chain saturated triglycerides and bile-related lipids. This pattern suggests that mule duck foie gras may represent a more neutral-lipid-concentrated deposition profile, whereas Landes goose foie gras may retain a broader structural and bile-associated lipid signature. Such an interpretation is consistent with previous work showing that overfeeding-induced hepatic steatosis in mule ducks is characterized by dynamic shifts in glucose utilization, lipid synthesis, cholesterol metabolism, and triglyceride storage, and that liver composition changes markedly as steatosis progresses (Chartrin et al., 2006; Mozduri et al., 2021; Pioche et al., 2020). More broadly, previous work suggests that mule ducks are typically associated with shorter, highly efficient foie gras production cycles, whereas geese are more often characterized by longer fattening periods and greater hepatic enlargement (Liu et al., 2016). In this context, the present results can be interpreted as reflecting distinct commercial foie gras types with different deposition patterns, rather than as evidence of strictly species-intrinsic compositional differences.

The results derived from different analytical approaches were systematically integrated, and compounds with relatively large fold changes and q < 0.05 were selected as representative differential molecules for further analysis. Their relative abundances in Landes goose and mule duck foie gras were subsequently compared in detail to provide complementary evidence for potential differences in metabolic characteristics between the two foie gras products.

**Fig. 6:**
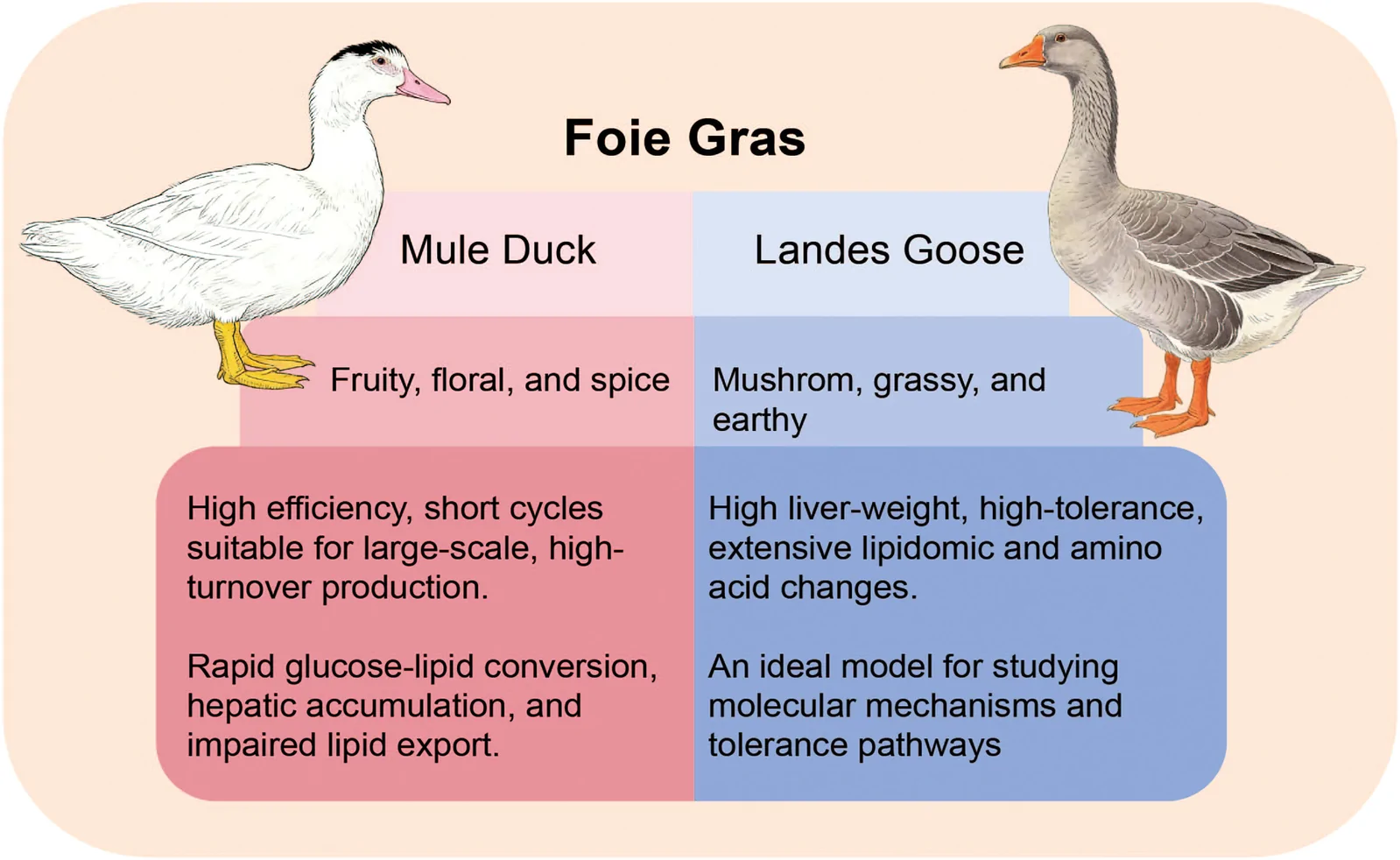
Overview of the major differential characteristics between mule duck and Landes goose foie gras.

From a mechanistic perspective, several differential molecules detected in this study may represent lipid-metabolism intermediates rather than merely compositional markers. For example, acylcarnitine-related metabolites are involved in mitochondrial fatty acid transport and β-oxidation, and their abundance may reflect differences in fatty acid utilization, mitochondrial lipid catabolism, or incomplete β-oxidation between foie gras types (Houten and Wanders, 2010; Dambrova et al., 2022). Bile acids and bile-related components are cholesterol-derived molecules that participate in lipid digestion, enterohepatic circulation, and lipid metabolic signaling (Chiang, 2009). In addition, DG, TG, PC, PE, PI, and PS species fall within glycerolipid and glycerophospholipid networks, which connect neutral lipid storage, membrane remodeling, and the availability of fatty-acyl precursors for oxidation-derived volatile formation (Fahy et al., 2009; van Meer et al., 2008). Therefore, the metabolomic and lipidomic differences observed here are better interpreted as a coordinated shift in lipid storage, lipid turnover, membrane-lipid organization, and lipid-derived volatile precursor pools, rather than as isolated differences in individual compounds. Pathway enrichment analysis provided an additional systems-level view of the differences between mule duck and Landes goose foie gras, but these results should be interpreted as annotation-based clustering of the integrated differential metabolite and lipid set rather than direct evidence of pathway activation. In particular, because metabolites and lipids were jointly used as input for enrichment analysis, the direction of pathway representation did not necessarily correspond exactly to the direction of individual compounds within a given pathway. Therefore, the appearance of bile acid-related pathways in the mule duck-enriched network should be interpreted cautiously as part of the overall pathway annotation pattern of the integrated differential set, rather than as direct evidence of increased bile acid biosynthetic flux. More broadly, the enrichment results support that the two foie gras types differ not only in individual compounds but also in their higher-order metabolic organization, especially with respect to phospholipid-related pathways in Landes goose foie gras and the broader nucleotide-, redox-, and lipid-related pathway representation in mule duck foie gras (Fig. 6).

An additional implication of this study is its potential value for product discrimination. Current authentication of foie gras products often relies on species identification methods, whereas the present results suggest that mule duck and Landes goose foie gras may also be differentiated using integrated chemical signatures from volatile compounds, polar metabolites, and lipid species. This provides a basis for future development of composition-based authentication tools.

Beyond product discrimination, the integrated compositional signatures identified in this study have potential value for commercial quality management and product standardization. Candidate markers derived from gross traits, volatile compound profiles, polar metabolites, and lipid species could be further validated to support product grading, processing-suitability assessment, flavor-related product characterization, and authenticity verification. In particular, combinations of markers associated with liver size and moisture, lipid deposition patterns, and volatile composition may help establish differentiated positioning of mule duck and Landes goose foie gras for distinct processing and market applications.

Nevertheless, several limitations should be acknowledged. First, because mule ducks and Landes geese were produced under different species-specific overfeeding durations (18 vs 29 d), the present design does not allow strict separation of species/genetic effects from production-regimen effects and cumulative overfeeding-duration effects. Second, the biological significance of differential metabolites and lipids was inferred from published literature rather than confirmed by targeted quantification or functional validation. Third, flavor-related conclusions remain chemical rather than sensory in nature. Future studies may therefore combine harmonized production conditions, such as time-matched or cumulative-feed-intake-matched overfeeding designs and serial sampling across comparable steatosis stages, with targeted validation and sensory or technological evaluation to better define the compositional and quality differences between foie gras types.

## Authors' Contributions

All authors made substantial contributions to the study, critically reviewed the manuscript for important intellectual content, approved the final version for publication, and agreed to be accountable for all aspects of the work and for ensuring that any questions related to the accuracy or integrity of the work are appropriately investigated and resolved.Shuisheng Hou, Zhengkui Zhou, and Dapeng Liu contributed to the conceptualization, coordination, and supervision of the study. Zhanbao Guo, Yi Tang, Jun Zhang, and Wei Min contributed to the selection and characterization of the experimental animal resources, the design and implementation of the animal experiment, and the interpretation of the associated phenotypic information. Zhengkui Zhou, Dapeng Liu, He Zhang, Yutong Xiao, Qiming Mu, Sidi Zhang, and Zhichao Hu contributed to the development and implementation of the experimental population and sampling protocols, phenotypic data collection, sample and data quality control, and data curation. Hehe Tang, Hongfei Liu, Sirui Liu, Jiaxin Zhang, Yongfu Zhang, and Lipeng Yuan contributed to data interpretation and the critical evaluation of the results. Dapeng Liu, Yutong Xiao, and Qiming Mu performed the bioinformatics and experimental analyses. Yutong Xiao drafted the manuscript. Zhengkui Zhou and Dapeng Liu reviewed and edited the manuscript. All authors participated in discussions concerning the experimental and analytical procedures and critically revised the manuscript for important intellectual content.

## Ethics approval and consent to participate

All experiments and protocols involved in animals were performed at the supervision of ethic regulation from the Institute of Animal Science, Chinese Academy of Agricultural Sciences (NO. IAS-2022-114), Beijing, China.

## Consent for publication

All authors have read and approved the final manuscript and have given their consent for its submission and publication in Poultry Science.

## Funding

This work was supported by the National Key R&D Program of China (2024YFF1000900), China Agriculture Research System of MOF and MARA (CARS-42-05), Scientific Research Project for Full-time Introduced High-end Talents of Hebei Province: Breeding and Extension of New High-quality and Disease-resistant Meat Duck Varieties (No. 2025HBQZYCXY017), Scientific Research Project for Full-time Introduced High-end Talents of Hebei Province: Creation of New Duck Germplasm and Research on New Variety Breeding (No. 2024HBQZYCXY031), and Key Institution for Academician Cooperation, Hebei Leshou Duck Industry Co., Ltd. (235A9915D).

## Ethics declaration

This study was conducted in accordance with the ARRIVE (Animal Research: Reporting of In Vivo Experiments) guidelines. This study was approved by the Institute of Animal Science, Chinese Academy of Agricultural Sciences. (Approval No. IAS‑2022‑114)

## Disclosures

The authors declare that they have no competing interests.

## Footnotes

Supplementary material associated with this article can be found, in the online version, at doi:10.1016/j.psj.2026.107681.
