# Supplementary material for: Comparative compositional analysis of commercially produced mule duck and Landes goose foie gras
Source: Poult Sci. 2026 Aug 23;105(12):107681. doi: 10.1016/j.psj.2026.107681 (PMC13594933; doi:10.1016/j.psj.2026.107681)
Supplement: Supplementary file 1 [file mmc1.docx]

Table 1: Ingredient composition and calculated nutrient levels of the experimental diets

| **Item** | **Duck** | **Goose** |
| --- | --- | --- |
| *Ingredient composition (%)* | | |
| Corn | 89.30 | 97.15 |
| Soybean meal | 3.85 | — |
| Soybean oil | 2.02 | — |
| Limestone | 0.29 | 0.50 |
| Dicalcium phosphate | 0.10 | — |
| Calcium bicarbonate | — | 0.35 |
| Vitamin-mineral premix | 0.10 | — |
| Vitamin AD3 premix | — | 0.30 |
| Duck oil | — | 1.00 |
| Salt | 0.34 | 0.50 |
| Functional additive | 4.00 | 0.20 |
| *Calculated nutrient levels* | | |
| Apparent metabolizable energy (AME), MJ/kg | 13.20 | 13.01 |
| Crude protein (CP), % | 9.42 | 7.38 |
| Ether extract (EE), % | 5.27 | 4.40 |
| Crude fiber (CF), % | 2.08 | 1.75 |
| Calcium (Ca), % | 0.16 | 0.29 |
| Total phosphorus (Total P), % | 0.28 | 0.31 |
| Lysine (Lys), % | 0.31 | 0.27 |
| Methionine (Met), % | 0.16 | 0.20 |

Note: —, not included or not listed; AME, apparent metabolizable energy; CP, crude protein; EE, ether extract; CF, crude fiber; Total P, total phosphorus; Lys, lysine; Met, methionine.
