# Supplementary material for: Comparative compositional analysis of commercially produced mule duck and Landes goose foie gras
Source: Poult Sci. 2026 Aug 23;105(12):107681. doi: 10.1016/j.psj.2026.107681 (PMC13594933; doi:10.1016/j.psj.2026.107681)
Supplement: Supplementary file 2 [file mmc2.docx]

**Figure S1**

**Volatile compounds with significantly differential abundance in Landes goose and mule duck foie gras.**

**Figure S2**

**Metabolomic compounds with significantly differential abundance in Landes goose and mule duck foie gras.**

**Figure S3**

**Lipidomic with significantly differential abundance in Landes goose and mule duck foie gras.**
